# Comparison of Laparoscopic and Robotic Intraoperative Adverse Events in Benign Gynecological Procedures and the Correlation of the Adverse Events With Postoperative Outcomes and Risk Analysis

**DOI:** 10.7759/cureus.80497

**Published:** 2025-03-12

**Authors:** Rooma Sinha, Bana Rupa, Rohit Raina, Moumita Bag

**Affiliations:** 1 Department of Gynecology/Minimal Access Surgery, Apollo Health City, Hyderabad, IND

**Keywords:** complications, intraoperative, laparoscopy, postoperative, robotic

## Abstract

Introduction

Intraoperative adverse events (iAEs) are a part of any gynecological surgery, including laparoscopy and robotic surgery. Robotic surgery, with advanced three-dimensional vision, is supposed to have fewer complications than laparoscopy. We aim to compare iAEs between laparoscopic (LA) and robotic (RA) procedures and correlate them with postoperative complications and risk factor analysis.

Methods

A prospective study was conducted in a tertiary care center between July and October 2023 with 80 cases (myomectomy, hysterectomy, and endometriosis resection). Intraoperative (as per ClassIntra classification) and postoperative (as per Clavien-Dindo classification) complications were recorded, and data was analyzed using IBM SPSS Statistics for Windows, Version 26 (Released 2019; IBM Corp., Armonk, New York, United States). Categorical variables were summarized as n (%), while quantitative variables were summarized by mean ±S.D. A p-value less than 0.05 was considered statistically significant. Multivariate logistic regression analysis was done for risk analysis (adjusted odds ratio with 95% confidence interval).

Results

Overall, we reported 3.75% (3/80) iAEs (all in LA) and 32.5% (26/80) postoperative complications. Increased postoperative complications were seen in LA 15/40 (37.5%) than in RA 11/40 (27.5%). However, the difference was statistically insignificant (p =0.12 and p =0.47). Cases with higher iAE grades consecutively experienced higher postoperative complications (p 0.0001). The independent clinical risk factors, ASA score II status (aOR: 2.335, 95%CI: 0.707-7.709), increasing uterine size (aOR: 1.076, 95%CI: 0.953-1.214), endometriosis (aOR: 2.337, 95%CI: 0.615-8.878), previous surgical history (aOR: 1.595, 95%CI: 0.544-4.677), and lower preoperative hemoglobin (aOR: 0.721, 95%CI: 0.502-1.036), affecting postoperative complications were analyzed. However, none of the factors had a statistically significant association with postoperative complications.

Conclusions

We observed lesser complication rates in robotic surgery than in conventional laparoscopy. We also found that those with higher iAE grades were strongly associated with higher postoperative complication grades.

## Introduction

With advancements in surgical techniques, complex surgical procedures are routinely performed by minimally invasive surgery (MIS) techniques. Gynecological surgeries that were traditionally performed by laparotomy are now also done by MIS. Intraoperative adverse events (iAEs), although not limited to a particular mode of surgery (laparoscopic or robotic), have a significant impact on the immediate postoperative period, long-term outcome, and quality of life. Robotic surgery with enhanced 3D vision, tremor filtration, and intuitive movements is considered a more precise and advanced surgical system; however, whether robotic assistance reduces intra and post-operative complications in surgery is still unanswered [1-3]. Compared to conventional laparoscopy, the robotic system also has unique potential risk factors for complications due to the operating surgeon’s position being remote from the operative field and lack of haptic feedback.

With the hypothesis that robotic surgery has fewer intraoperative complications due to advanced technology, the objective of this study was to document and compare various intraoperative complications between the two MIS procedures using a validated system. Unfortunately, an iAE is neither clearly defined nor reported well during surgery [4], whereas reporting of postoperative complications is already standardized using the Clavien-Dindo classification system [5]. For reporting intraoperative complications, we used the ClassIntra classification system, published in 2020. The classification defines an iAE as any deviation from the ideal intraoperative course that occurs between skin incision and skin closure [6].

In this study, we aim to compare intraoperative complications between the two MIS procedures, correlate iAEs with postoperative complications, correlate demographic and surgical characteristics with intraoperative and postoperative complications, and perform a risk analysis of various preoperative factors. The findings of the study will thus help in selecting a safer surgical tool, enhancing patient safety, and reducing long-term morbidity.

## Materials and methods

We performed a prospective cohort study at Apollo Health City, Jubilee Hills, Hyderabad, India, after obtaining institutional ethics committee approval (IEC No: AHJ-ACD-012/05-23 dated 28th July 2023) from July 2023 to October 2023. The study population was women aged 20-60 years who underwent MIS surgery for benign gynecological conditions by an MIS-experienced single surgeon. The inclusion criteria were (a) total laparoscopic hysterectomy, (b) laparoscopic myomectomy, (c) laparoscopic endometriosis excision (LEE), (d) robotic hysterectomy (RH), (e) robotic myomectomy (RM), and (f) robotic endometriosis excision (REE). Any surgery done for recurrence or malignancy was excluded. Written consent was taken from all patients. The study population was divided into the laparoscopic group (LA) and the robotic group (RA), and demographic details were recorded. Open Hassan’s entry technique was followed in all cases. Laparoscopic surgery was done using bipolar forceps, harmonic scalpel, and laparoscopic scissors, while robotic surgery was performed using da Vinci Xi using two arms and three instruments (fenestrated bipolar forceps, hot shears (monopolar), and mega needle holder). For myomectomy cases, inj. vasopressin (1: 200) was used for reducing intraoperative hemorrhage as a standard technique. Suturing of the myometrium or the vault was done using barbed suture (Vloc) no 0 in all cases. For large specimens, the tissue retrieval technique was cold knife morcellation in an endo-bag in both arms. Endometriosis staging was done as per the American Association of Gynecologic Laparoscopists (AAGL) classification. Enhanced Recovery After Surgery (ERAS) protocol was followed for all preoperative, intraoperative, and postoperative phases [7]. Total operative time was calculated from skin incision to closure. All iAEs were reported as per the ClassIntra classification (Table 1), and all postoperative complications occurring within 30 days were reported as per the Clavien-Dindo classification (Appendices).

**Table 1:** ClassIntra classification of intraoperative adverse events

Grade	Definition	Examples
0	No deviation from the ideal intraoperative course	------
I	Any deviation from the ideal intraoperative course: a) Without the need for any additional treatment or intervention and b) Patient with no or mild symptoms	Bleeding: bleeding above average from small caliber vessel, self-limiting or definitively manageable without additional treatment than routine coagulation. Injury: minimal serosal intestinal lesion, not requiring any additional treatment. Cautery: small burn of the skin, no treatment necessary. Arrhythmia: arrhythmia (e.g. extrasystoles) without relevance
II	Any deviation from the ideal intraoperative course: a) With the need for any additional minor treatment or intervention and b) Patient with moderate symptoms, not life threatening, and not leading to permanent disability	Bleeding: bleeding from medium caliber artery or vein, ligation; use of tranexamic acid. Injury: non-transmural intestinal lesion requiring suture(s) Cautery: moderate burn requiring non-invasive wound care Arrhythmia: arrhythmia requiring administration of antiarrhythmic drug, no hemodynamic effect
III	Any deviation from the ideal intraoperative course: a) With the need for any additional moderate treatment or intervention and b) Patient with severe symptoms, potentially life threatening or potentially leading to permanent disability	Bleeding: bleeding from large caliber artery or vein with transient hemodynamic instability, ligation or suture; blood transfusion Injury: transmural intestinal lesion requiring segmental resection. Cautery: severe burn requiring surgical debridement Arrhythmia: arrhythmia requiring administration of antiarrhythmic drug, transient hemodynamic effect
IV	Any deviation from the ideal intraoperative course: a) With the need for any additional major and urgent treatment or intervention and b) Patient with life-threatening symptoms or leading to permanent disability	Bleeding: life-threatening bleeding with splenectomy; massive blood transfusion; stay at intensive care unit. Injury: injury of central artery or vein requiring extended intestinal resection. Cautery: life-threatening burn injury by cautery leading to fire requiring intensive care treatment. Arrhythmia: arrhythmia requiring electro-conversion, defibrillation, or admission to intensive care
V	Any deviation from the ideal intraoperative course with intraoperative death of the patient	----------

The sample size was calculated by using the formula N= Z 2PQ/L2 where N: sample size, Z: Z score 1.96 for a confidence level (CI) of 95%, P: prevalence, Q: 1-P, and L: margin of error. A study by Dell-Kuster et al. (2020) reported 24% experienced at least one iAE [6]. So, using the values Z=1.96, P=24, Q=100-24=76%, and L=10%, the sample size was calculated as N= 1.96×1.96 ×24×76/10×10. Making it to near value, a minimal sample size of 70 was required. We considered our sample size to be 80, with an equal distribution of 40 cases in both arms.

Data entry was done using Microsoft Excel 2010 (Microsoft Corporation, Redmond, USA), and statistical analysis was done using IBM SPSS Statistics for Windows, Version 26 (Released 2019; IBM Corp., Armonk, New York, United States). Descriptive statistical analysis was done, and categorical variables were summarized with n (%), while quantitative variables were summarized by mean ± S.D (Standard deviation). A p-value less than 0.05 was considered statistically significant. Analysis of risk factors associated with intraoperative and 30-day postoperative complications was done by applying multivariate logistic regression analysis to calculate the adjusted odds ratio (aOR) with a 95% confidence interval (CI).

## Results

Eighty cases were included in this study, 40 cases in each arm. Thirty-three patients underwent hysterectomy (LH: 20; RH: 13), 26 patients underwent myomectomy (LM: 10; RM:16), and 21 patients underwent endometriosis excision surgery (LEE: 10; REE: 11). Associated features of endometriosis were seen in three cases of hysterectomy (LH: 2; RH: 1) and four cases of myomectomy (LM: 2; RM: 2). Demographic profiles of both groups were comparable, i.e., age, BMI, ASA (American Society of Anesthesiologists) score, preoperative hemoglobin, and previous abdominal surgeries (Table 2).

**Table 2:** Demographic characteristics SD: standard deviation; n: number; BMI: body mass index; ASA: American Society of Anesthesiologists; AUB: abnormal uterine bleeding; AUB-L: leiomyoma; AUB-A: adenomyosis; AUB-E: endometrium.

S. No.	Baseline characteristics	Mean ±SD		p-value
		Laparoscopic	Robotic	
1	Age (years)	39.70±9.14	36.18±6.88	0.055
2	BMI (kg/m^2^)	26.47±5.20	25.86±4.35	0.572
3	Uterine size (weeks)	11.55±4.82	13.40±5.64	0.102
4	Hemoglobin (gm/dl)	11.74±1.73	11.86±1.47	0.740
		% (n)		
		Laparoscopic	Robotic	
5	ASA score			0.13
	ASA I (n=58)	65% (26)	80% (32)	
	ASA II (n=22)	35% (14)	20% (8)	
6	Charlson comorbidity index			0.18
	0	92.5% (37)	80% (32)	
	1	7.5% (3)	15% (6)	
	2	0	5% (2)	
7	Previous surgery history	33.5% (13)	40% (16)	0.48
		n		
8	Indication of hysterectomy	Laparoscopic	Robotic	
	AUB-L	10	11	
	AUB-A	6	1	
	AUB-E	4	1	

Various intraoperative and postoperative characteristics are shown in Table 3 and Table 4.

**Table 3:** Intraoperative characteristics SD: standard deviation

S. No.	Characteristics	Mean ±SD		p-value
		Laparoscopic	Robotic	
1	Blood loss (ml)	139.25±189.04	145.88±162.48	0.589
2	Operative time (min)	118.75±51.92	122.75±27.19	0.06
3	Postoperative stay (hours)	39.48±37.20	39.58±21.62	0.724

**Table 4:** Intraoperative and postoperative characteristics AAGL: American Association of Gynecologic Laparoscopists; n: number

S. No.	Characteristics	n (%)		p-value
		Laparoscopic	Robotic	
1	AAGL stage			
	<2	5 (27.8%)	2 (12.5%)	0.40
	≥2	13 (72.2%)	14 (87.5%)	
2	iAE			
	No	37 (92.5%)	40 (100%)	0.12
	Yes	3 (7.5%)	0 (0%)	
3	Postoperative complications			
	No	25 (62.5%)	29 (72.5%)	0.47
	Yes	15 (37.5%)	11 (27.5%)	

In endometriosis cases, though AAGL stage (≥2) was slightly higher in the RA arm (87.5%; n=14) than in LA (72.2%; n=13), it was not statistically significant (p =0.40). We reported a 7.5% (3/40) iAE in the laparoscopic arm and 0% iAE in the robotic arm according to the ClassIntra classification, and 26/80 (32.5%) postoperative complications as per the Clavien-Dindo classification (Table 5).

**Table 5:** Case details: intraoperative and postoperative complications and their management LM: laparoscopic myomectomy; LH: laparoscopic hysterectomy; LEE: laparoscopic endometriosis excision; RM: robotic myomectomy; RH: robotic hysterectomy; REE:  robotic endometriosis excision; iAEs: intraoperative adverse events; PRBC-packed red blood cells; POD: postoperative day.

S.No	Procedure	iAE	iAE grade (ClassIntra)	Management	Operative time (Mins)	Postop-complication grade (Clavien Dindo)	Management	Length of stay (hrs)
1	LM	----	----	----	90	I	I.V analgesic	24
2	LH	----	----	----	210	II	I.V antibiotic, analgesic	72
3	RM	----	----	----	240	II	1 PRBC transfused	24
4	RM	----	----	----	175	I	I.V analgesic	48
5	LM	----	----	----	100	I	I.V analgesic	24
6	REE	----	----	----	120	I	I.V antibiotic, analgesic	96
7	LM	----	----	----	180	I	I.V analgesic	48
8	LEE	Rectal mucosal injury	III	Laparoscopic suturing	180	II	I.V antibiotic, analgesic	120
9	LM	----	----	----	120	I	I.V analgesic	48
10	LH	Inadvertent cystostomy	III	Laparotomy and repair	240	II	I.V antibiotic, analgesic	144
11	REE	----	----	----	150	I	I.V analgesic	24
12	LM	----	----	----	60	I	I.V analgesic	24
13	LEE	----	----	----	170	I	I.V antibiotic, analgesic	36
14	LH	----	----	----	75	I	I.V analgesic	24
15	RM	----	----	----	210	II	1 PRBC transfused	24
16	LH	----	----	----	110	I	I.V analgesic	24
17	LH	----	----	----	95	I	I.V analgesic	24
18	REE	----	----	----	150	I	I.V antibiotic, analgesic	48
19	RM	----	----	----	120	I	I.V antibiotic, analgesic	48
20	RM	----	----	----	270	II	1 PRBC transfused	48
21	LM +LEE	----	----	----	235	II	I.V antibiotic, analgesic	48
22	RH	----	----	----	145	I	I.V analgesic	96
23	REE	----	----	----	150	I	I.V antiemetic, analgesic	48
24	LM	Sigmoid colon serosal tear	II	Laparoscopic Suturing	240	II	I.V antibiotic, analgesic 2 PRBC transfused	72
25	REE	----	----	----	150	III b (fever, abdominal pain, obstipation, no nausea vomiting)	Reoperation on 8^th^ POD (exploratory laparotomy + sigmoid colon repair + diversion loop ileostomy)	96
26	LM	----	----	----	210	IIIb (epigastric pain, nausea vomiting, no fever)	Reoperation on 10^th^ POD (diagnostic laparoscopy + bowel adhesiolysis)	72

Intraoperatively, we had an unintentional bladder injury in one laparoscopic case (LH), which occurred during adhesiolysis (previous surgery) and was managed by laparotomy (conversion) and bladder repair. Two cases of bowel injury were encountered; one case of LM had a sigmoid colon serosal injury, and another case of LEE surgery had a rectal injury. Both cases were managed by intraoperative laparoscopic suturing. There was no need for intraoperative conversion.

We also observed postoperative complications. Grade I complications (pain, fever, nausea, vomiting, shoulder tip pain, and urinary complaints) were seen in 20% of cases (16/80) and were managed by intravenous analgesic and antiemetics (LA 9, RA 7). Grade II postoperative complications were reported in 10% of cases (8/80) (LA 5, RA 3). Blood transfusion was required in four cases (1 LM, 3 RM), whereas Grade III b complications were seen in 2.5% of cases (2/80) (LA 1, RA 1). Two cases of bowel injury presented as post-operative complications with signs and symptoms of intestinal obstruction and got reoperated. One case of laparoscopic myomectomy presented on the 10th postoperative day and was managed by laparoscopic adhesiolysis of the bowel loops from the myomectomy scar. Another case of robotic endometriosis excision surgery had laparotomy and sigmoid colon repair on the eighth postoperative day. The readmission and reoperation rates were equal in both arms and insignificant (p-value: 0.971). One case had a postoperative stay in the ICU in the LA arm because of medical co-morbidities.

Although we had all iAEs (7.5% (3/40) in LA vs 0 % in RA) and more postoperative complications in the LA group than in the RA group (37.5% (15/40) vs 27.5% (11/40)), the difference was not statistically significant (p =0.12 and p =0.47). We found that patients with intraoperative complications (≥ Grade II ClassIntra) also experienced higher postoperative complications (Grade II Clavien-Dindo). The increasing severity of iAEs significantly correlated with the occurrence of >grade II postoperative complications (p-value <0.0001; highly significant) (Table 6).

**Table 6:** Relationship of iAEs (ClassIntra) with postoperative complications (Clavien-Dindo classification) iAE: intraoperative adverse event; n: number.

iAE (Yes/No)	Postoperative grade (n, %)				Total	p-value
	0	1	2	3		
No	54 (70.1%)	16 (20.8%)	5 (6.5%)	2 (2.6%)	77	
Yes	0	0	3 (100%)	0	3	< 0.0001
Total	54 (67.5%)	16 (20%)	8 (10%)	2 (2.5%)	80	

However, a grade IIIb Clavien-Dindo was seen in 2.56% (2/78) of cases without iAEs. Multivariable logistic regression analysis was done for postoperative complication risk factor analysis (Table 7).

**Table 7:** Multivariate logistic regression analysis for factors associated with postoperative complications Hb: hemoglobin; ASA: American Society of Anesthesiologists.

S. No	Characteristics	p-value	Adjusted odds ratio	95% CI for odds ratio	
				Lower	Upper
1	Age (years)	0.706	0.984	0.908	1.068
2	Hb (preoperative)	0.077	0.721	0.502	1.036
3	Uterine size (weeks)	0.238	1.076	0.953	1.214
4	ASA grade II status	0.164	2.335	0.707	7.709
5	Endometriosis	0.213	2.337	0.615	8.878
6	Previous surgical history	0.395	1.595	0.544	4.677
7	Laparoscopic surgery	0.230	2.136	0.619	7.369

The independent clinical risk factors affecting postoperative complications were analyzed, ASA score II status (aOR:2.335, 95%CI: 0.707-7.709), increasing uterine size (aOR:1.076, 95%CI: 0.953-1.214), endometriosis (aOR:2.337, 95%CI: 0.615-8.878), previous surgical history (aOR:1.595, 95%CI: 0.544-4.677), lower preoperative hemoglobin (aOR:0.721, 95%CI: 0.502-1.036) and, laparoscopic surgery (mode of surgery) (aOR:2.136, 95%CI: 0.619-7.369). However, none of the factors had a statistically significant association with postoperative complications.

## Discussion

We did a comparative analysis of various iAEs between two minimally invasive gynecological surgery techniques. The study included three broad categories of MIS gynecological surgeries, i.e., hysterectomy, myomectomy, and endometriosis excision surgery with a comparable demographic characteristic. It is the first study from India to compare two MIS approaches, i.e., laparoscopic and robotic.

The lack of a standardized definition for iAEs in gynecology made reporting intraoperative complications difficult. In a systematic review by Watrowski et al. (2021), a wide range of intraoperative and postoperative complication rates were seen due to variable definitions of iAEs, grading systems, inter-institutional variability, and differences between individual surgeons (laparoscopic 0.2% -18% and robotic 3.2% -14.6%) [4]. We used the ClassIntra classification system for reporting iAEs, which is the first prospectively validated classification system for assessing iAEs in a standardized way. The classification, though not specific to gynecology, also links iAEs to the well-established Clavien-Dindo classification of postoperative complications.

Complications commonly noted in robotic gynecology surgery are primary port site entry [8], bleeding, bladder, ureter, bowel injury [9], and conversion, which are also seen in laparoscopy. We used the modified Hassan technique for creating primary ports in both the arms and didn’t have any complications during this step, even in cases where dense adhesions of previous surgery were present. Though none of the known techniques is superior [10], mastering any one technique and following the same gives the best result.

A robotic system with precise maneuvers leads to better control of bleeding from small vessels [11] and offers superiority over laparoscopy in managing vascular injuries [12]. However, in our study, we did not observe a significant difference in blood loss in the two arms (p 0.589).

Bladder injury (0.24%) is commonly seen in hysterectomy cases during adhesiolysis of the bladder in LH [13]. The robotic surgery precision and 3D vision are said to help in precise adhesiolysis, minimizing bladder complications. However, Petersen et al. (2018) in a retrospective study of hysterectomies found a similar rate of urologic injury (0.92% in robotic and 0.90% in laparoscopic hysterectomy) [14]. We observed an intraoperative bladder injury rate of 2.5% (1/40), which occurred during bladder adhesiolysis in a case of LH and required laparotomy for repair.

In our study, intraoperative bowel injury was seen in two cases, both in LA (5%,2/40), and was repaired laparoscopically without any conversion. The bowel injury rate in systematic reviews of robotic gynecologic surgery was 0.62% [15], and in the case of laparoscopic gynecologic surgery, it was 0.13% [16]. Both studies highlighted inconsistent definitions of bowel injury and didn’t specify bowel injury (serosal injury, enterotomy, or perforation), causing either over or underreporting of the injuries. In our study, while recording bowel injury, we did mention the location, extent, and subsequent management of bowel injury to obtain a real incidence of bowel injury while comparing the two MIS arms.

Conversion during a surgery should never be seen as a failure; instead, it should be seen as a procedural change for patient safety, and it should be clearly defined whether it is due to any complication or due to deviation from a planned approach. We had a single case of conversion to laparotomy due to bladder injury during LH (2.5%,1/40). Similar to our study findings, Fotiou et al. (2021) suggested superior management of bleeding robotically rather than laparoscopically, avoiding conversion to laparotomy with the rapid achievement of hemostasis by robotic suturing [17]. A higher rate of conversion was seen in robotic surgery as compared to laparoscopy when differentiation between the cause of conversion was not done, and deviation from the planned approach was also included in conversion rates [18].

An increase in operative time is seen in the case of intraoperative complications depending on the location and severity [6]. Although we found an increase in the operative time (1.5 times) in cases with iAEs, it was not statistically significant, which highlights the effective handling of complications by an experienced surgeon.

Overall, we reported 32.5% (26/80) postoperative complications according to the Clavien-Dindo Classification. Grade I postoperative complications (Clavien-Dindo classification) were observed in 20% of the cases. These cases did not require any deviation from the routine postoperative management. We had two cases (1 in LA and 1 in RA) of bowel injury presented and recognized postoperatively. We believe an electrosurgical device (ESD)-related injury was the reason for the delayed presentation of the injury. Delayed presentation and recognition are often seen when the injury is related to an ESD distant from the surgeon’s field of view. Bowel injury is a potential complication in MIS, and intraoperative recognition is crucial for prompt management and improved outcomes. If one is vigilant, most ESD-related complications are preventable [19]. Precision surgery, aided by a robotic system, reduces bowel complications, but ESD, which is surgeon error, can happen in both platforms of MIS. So, one needs to be careful while using ESD.

Though we had more complications in LA than RA, we did not find any statistical difference between the two groups consistent with previous studies [9,20,21]. Robotic surgery done by a high-volume experienced gynecologist experienced fewer intraoperative complications and significantly fewer postoperative complications [21,22]. Our study's low rates of complications could reflect the surgeon’s high case volume (both laparoscopic and robotic), a consistent team, and the use of a consistent, standardized surgical technique and equipment.

While considering the impact of iAEs on postoperative complications, we found a significant correlation between increasing iAE grade and the occurrence of higher-grade postoperative complications (p< 0.0001), and an increase in the length of postoperative stay (3 times) was also seen in cases with iAE more than grade 2 highlighting the importance of following surgical safety principles.

We also analyzed the risk factors associated with postoperative complications using multivariable logistic regression. Demographic profiles of both groups (LA vs RA) in our study were comparable, including age, BMI, ASA score, preoperative hemoglobin, and previous abdominal surgeries, which avoided any bias in terms of the complexity of the surgery. Although the ASA classification has not been designed as a predictive index of perioperative risk, it can be used as an excellent index to estimate the risk in gynecological surgery [23], and a greater risk of postoperative complications is seen in cases with ASA score II than ASA score I [24]. Previous abdominal surgery and the presence of endometriosis are associated with intraabdominal adhesions, which make these surgeries more complex. Preoperative anemia, especially seen in older women, is an independent risk factor for adverse postoperative outcomes [25]. Casarin et al. (2021) in their study performed multivariable analysis and found that endometriosis (odds ratio: 3.51; p = 0.02), intraoperative complication (odds ratio: 3.10, p < 0.001) and conversion to open surgery (odds ratio: 1.26, p < 0.001) were independent risk factors for major postoperative complications [26]. Similarly, in a retrospective study by Le et al. (2023), previous abdominal surgery (aOR: 3.58, 95% CI: 1.38-6.54), advanced age (>60 years-old vs. 18-30 years-old: aOR: 2.92, 95% CI: 1.67-5.65), obesity (aOR: 2.52, 95% CI: 1.39-7.28) and the type of surgical procedure were the primary factors associated with major complications in gynecologic laparoscopic surgery [27].

Though not statistically significant, we found a higher aOR for the occurrence of postoperative complications in cases with ASA score II status, presence of endometriosis, history of previous surgery, and laparoscopic surgery as a mode of surgery. The higher grades of intraoperative complications also correlated well with higher grades of postoperative complications, suggesting that minimizing or avoiding iAEs will lead to lower grades of postoperative complications. Identifying preoperative risk factors for complications can help improve patient outcomes by reducing complications. Less intraoperative and postoperative complications could translate to a reduced incidence of additional surgical or medical interventions, shorter recovery, and shorter hospital stays, reducing the overall economic burden of treatment.

Our study compared iAEs in laparoscopic and robotic assisted minimally invasive gynecologic surgeries and highlighted the lack of standardized definitions affecting complication reporting. While robotic surgery showed fewer complications, offering advantages with precision and effective complication management, no statistically significant differences were found between the two approaches, emphasizing the importance of surgical expertise, standardized techniques, and identifying preoperative risk factors to improve patient outcomes.

Strengths and limitations of the study

This is a prospective study to report iAEs in MIS gynecology, according to the ClassIntra classification system, and compare the association of iAEs with an already validated postoperative complications grading system (Clavien-Dindo). The major limitation of the study is that it is a single center and single surgeon; however, the surgical technique followed is simple, standardized, and easily reproducible, which allows for the generalization of the findings.

## Conclusions

During any surgery, practicing safe surgical techniques to avoid surgical complications should be the aim. However, if complications do occur, one should report and document them, regardless of whether minor or major. Due to the lack of any standardized reporting system in gynecology at present, we felt a strong need to clearly define intraoperative complications. While not statistically significant, our study found lower complication rates in robotic surgery compared to conventional laparoscopic surgery when performed by experienced surgeons, highlighting the necessity of having well-trained robotic gynecologists. We also found a strong association between higher iAE grades and higher postoperative complication grades, emphasizing the importance of surgical safety on recovery. Thus, robotic surgery can be used to minimize iAEs and concurrent postoperative complications. We also recommend a detailed preoperative risk assessment to further reduce intraoperative and postoperative complications and help predict postoperative outcomes during patient counseling. We suggest that this study be considered a pilot study, as it can be used as a foundation for a more extensive multicenter study.

## Appendices

**Table 8:** Postoperative complications according to Clavien-Dindo classification

Grade	Definition
Grade I	Any deviation from the normal postoperative course without the need for pharmacological treatment or surgical, endoscopic, and radiological interventions Allowed therapeutic regimens are drugs as antiemetics, antipyretics, analgesics, diuretics, electrolytes, and physiotherapy. This grade also includes wound infections opened at the bedside.
Grade II	Requiring pharmacological treatment with drugs other than such allowed for grade I complications. Blood transfusions and total parenteral nutrition are also included.
Grade III	Requiring surgical, endoscopic or radiological intervention
Grade III a	Intervention not under general anesthesia
Grade III b	Intervention under general anesthesia
Grade IV	Life-threatening complication (including CNS complications) requiring IC/ICU management
Grade IV a	Single organ dysfunction (including dialysis)
Grade IV b	Multiorgan dysfunction
Grade V	Death of a patient
